# Luminescent Phage-Based Detection of *Klebsiella pneumoniae*: From Engineering to Diagnostics

**DOI:** 10.3390/ph14040347

**Published:** 2021-04-09

**Authors:** Lior Zelcbuch, Elad Yitzhaki, Olga Nissan, Eliya Gidron, Nufar Buchshtab, Edith Kario, Sharon Kredo-Russo, Naomi B. Zak, Merav Bassan

**Affiliations:** Research Department, BiomX Ltd., Ness Ziona 7414002, Israel; Elad3339@gmail.com (E.Y.); alechka48@gmail.com (O.N.); eliyag@biomx.com (E.G.); nufarb@biomx.com (N.B.); edithk@biomx.com (E.K.); sharonk@biomx.com (S.K.-R.); naomiz@biomx.com (N.B.Z.); meravb@biomx.com (M.B.)

**Keywords:** diagnostics, phage, luminescent, cloning, *nluc*, feces

## Abstract

Bacteriophages (“phages”) infect and multiply within specific bacterial strains, causing lysis of their target. Due to the specific nature of these interactions, phages allow a high-precision approach for therapy which can also be exploited for the detection of phage-sensitive pathogens associated with chronic diseases due to gut microbiome imbalance. As rapid phage-mediated detection assays becoming standard-of-care diagnostic tools, they will advance the more widespread application of phage therapy in a precision approach. Using a conventional method and a new cloning approach to develop luminescent phages, we engineered two phages that specifically detect a disease-associated microbial strain. We performed phage sensitivity assays in liquid culture and in fecal matrices and tested the stability of spiked fecal samples stored under different conditions. Different reporter gene structures and genome insertion sites were required to successfully develop the two *nluc*-reporter phages. The reporter phages detected spiked bacteria in five fecal samples with high specificity. Fecal samples stored under different conditions for up to 30 days did not display major losses in reporter-phage-based detection. Luminescent phage-based diagnostics can provide a rapid co-diagnostic tool to guide the growing field of phage therapy, particularly for a precision-based approach to chronic diseases treatment.

## 1. Introduction

Pro-inflammatory microenvironments are triggered by microbial colonization and immune responses to bacteria. The gut microbiome has thus emerged as a significant factor in the management or prevention of chronic inflammatory diseases. For example, *Helicobacter pylori* is known to induce inflammation and cause a spectrum of gastric diseases including gastritis, ulcers, and gastric cancer [1]. *Klebsiella pneumoniae* causes a wide range of infections such as urinary tract infections, and is also thought to be involved in inflammatory bowel and liver diseases [2,3,4].

Although antibiotics have been used for decades as a powerful treatment modality for infections in both human therapy and animal husbandry, antibiotic use not only promotes the development of antibiotic-resistant bacterial strains but also leads to adverse outcomes such as alteration of the population of beneficial commensal gut microbiota and host immunity, making this therapeutic modality inappropriate for use in bacterial-pathogen-associated chronic inflammatory diseases. Because of the recognition of the harms that these broad-spectrum therapeutics exert on the beneficial commensal microbiome, and because of the effect of prolonged antibiotic use on antibiotic resistance, scientists have resumed their efforts in the search for alternative approaches to treating diseases and chronic conditions associated with pathogenic bacterial colonization, with a particular interest in methods that allow precise targeting of the culprit bacteria [5,6,7,8,9,10].

One such alternative treatment option is bacteriophage therapy, which was previously overshadowed by antibiotic therapy. Bacteriophages (“phages”, viruses that infect specific bacteria) are considered natural enemies of bacteria with high species and even strain specificity and the ability to self-amplify precisely where required [11]. Due to their orthogonal mode of action, they are impervious to the antibiotic-resistant state of their target bacteria. Additionally, their narrow host range and minimal off-target effects ensure that the integrity of the beneficial commensal microbiome is preserved [12]. The specific nature of phage–host interactions allows for a precision-medicine approach while optimizing the efficacy of phage therapy. However, the precision approach necessitates the prior evaluation of the phage susceptibility of the target pathogenic bacterial strain or strains found in the patient in order to determine their suitability for the proposed treatment.

Currently, the most common methods of determining bacterial susceptibility to a specific phage require isolation of the bacteria of interest followed by screening for the phage sensitivity of the isolate using either the classic double-layer overlay assay (the current gold standard) or liquid infection assays [13,14,15]. Although both methods are easy to implement in any laboratory setting at a low cost, they are very time-consuming—both because of their reliance on the isolation of the pathogenic bacterial strain and the requirement for long incubation times to yield a measurable response. These factors make them less suitable for use as standard procedures [13].

Bacteriophage genome engineering offers the possibility of streamlining the process for determining the phage sensitivity of a target bacteria by making the bacterial isolation step redundant [16,17]. In this approach, currently being implemented primarily in the food industry to detect the presence of microbial contamination, engineered bioluminescence-based reporter bacteriophage assays offer the best available method for determining the presence of specific bacteria with a fast turnaround time and without the need for bacterial isolation [18,19]. Accordingly, reporter phage assays are good candidates for serving as diagnostic tools in the clinical setting. In this approach, *nluc*, a gene encoding the NanoLuc luciferase bioluminescent protein, is cloned into the bacteriophage genome in such a manner that it is expressed only after phage infection of its target bacteria. At that time, with the addition of substrate, the infected bacterium emits a bioluminescent signal, verifying the expression of the *nluc* gene encoded in the phage genome [20].

**Figure 1:**
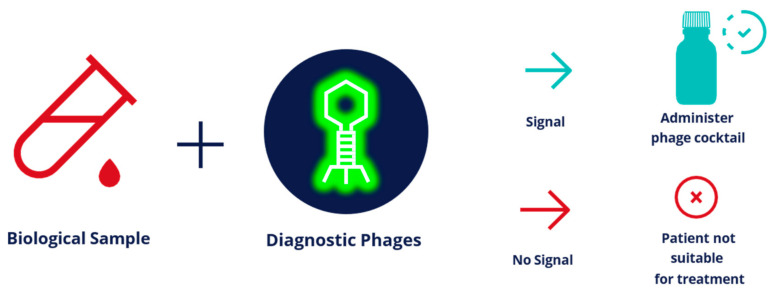
A schematic model of phage-based diagnostics. The biological sample is incubated with the luminescent phage cocktail; the detection of a luminescent signal validates the presence of the target bacteria in the sample. This ensures that the administration of the cocktail will result in phage activity within the patient. If no luminescence signal is detected, the target bacteria are not present in the sample (and therefore in the patient), hence the cocktail is considered not to be suitable for treatment.

NanoLuc-based reporter phages have demonstrated a faster turnaround time than standard culture-dependent approaches by enabling the direct identification of bacterial infection within an unprocessed sample without the need for tedious steps of bacterial isolation (Figure 1) [18,21]. These characteristics also make the approach potentially amenable to the development of a point-of-care kit facilitating the on-site evaluation of patient suitability for phage-enabled precision therapy. In the precision therapy approach, a fixed cocktail of approximately three to eight phages is designed against a pathobiont bacterial strain or collection of clinical strains and is administered to patients found to be colonized with microbes that are susceptible to the designed cocktail. This phage therapy approach offers the advantages of providing an “off the shelf” product that easily fits into the present regulatory framework and readily addresses a broad patient population, in contrast to a personalized phage therapy approach in which a large panel of phages are tested against an individual patient’s bacteria to identify a therapeutic phage, which sometimes necessitates the isolation of new phages. In addition, current methods describing the insertion of the luminescence gene into a phage genome are not always successful in yielding the required sensitivity. Extending the toolkit for the engineering of reporter phages will assure that this approach can be applied to many more potentially therapeutic phages.

In this report, we describe different methods used in the engineering of two phages that comprise part of a phage cocktail targeting a specific pro-inflammatory *Klebsiella pneumoniae (KP)* strain, KP2H7, found in the feces of many inflammatory bowel disease (IBD) patients. The luminescent phages will enable the direct assessment of patient fecal samples for the presence of this strain without the need for bacterial isolation. This will support a precision-medicine approach for the treatment of IBD patients that are colonized with KP2H7 shown to exacerbate the symptoms of IBD. Additionally, we examine the sensitivity of the bioluminescent assay using the engineered reporter phages—specifically, the lowest target bacterial count in fecal samples that are detectable by this assay. We also demonstrate the stability of the fecal bacterial population under different fecal storage conditions. This observation supports the possibility of delayed testing, as is likely to be required in clinical settings, and thereby makes a precision approach to phage treatment more feasible.

## 2. Results

Phage engineering can be accomplished by various methods, such as using cell-free in-vitro transcription translation systems that also enable the assembly of phage genomes [22] or phage “rebooting” using *Saccharomyces cerevisiae* [23] or L-form bacteria [24]. In this study, we used the traditional approach of homologous recombination to knock-in the *nluc* gene into the genome of wildtype phages in such a way that it is expressed only upon target bacteria infection [20].

### 2.1. Molecular Cloning of NanoLuc into Mcoc and 8M7 Bacteriophages

For engineering, we used two therapeutic phages from different families: Mcoc (a Drulisvirus phage within the family Podoviridae with a 44 kb genome) and 8M7 (a phage within the family Siphoviridae with a 115 kb genome). These two phages are included in the BiomX BX002 phage cocktail for the treatment of IBD, targeting KP2H7. Engineering of these phages has been carried out to allow screening for the presence of phage-sensitive KP2H7 in candidates for inclusion in a planned clinical trial and subsequent treatment.

The first step in the engineering of a bioluminescence-based reporter phage is to identify a highly expressed phage protein so that its promoter can be used to drive the expression of the introduced *nluc* gene. The major capsid protein is known to be highly expressed; therefore, using genome annotation algorithms and confirmation by liquid chromatography/mass spectrometry (LC/MS) [25,26,27], the major capsid proteins of Mcoc and 8M7 were determined.

**Figure 2:**
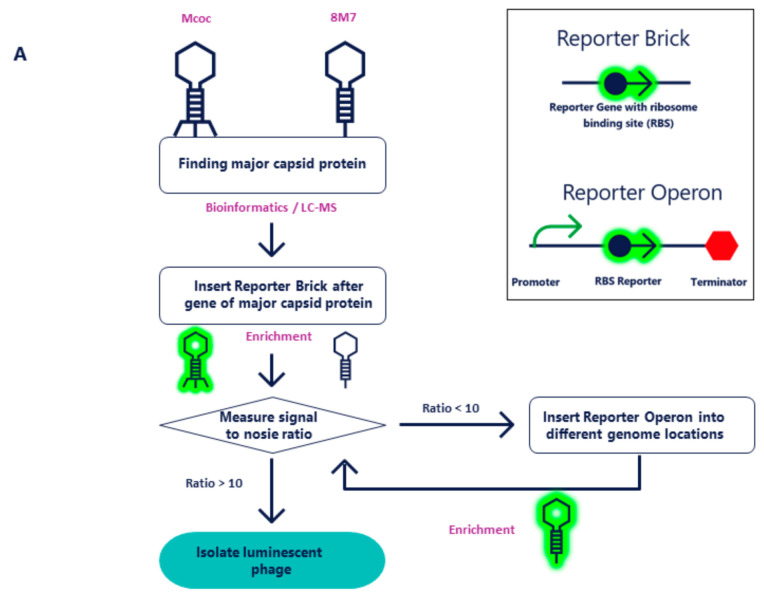

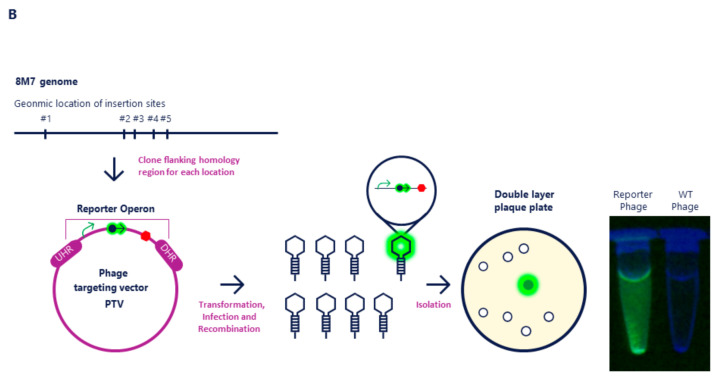
The Reporter Brick and Reporter Operon phage engineering design and workflow. (**A**) The overall workflow of the engineering method. Mcoc phage represents the standard approach in which adding the Reporter Brick after the major capsid protein is sufficient to attain a luminescent phage of high sensitivity. Phage 8M7 represents the alternative strategy employed when the standard approach does not produce a highly sensitive luminescent phage. In this strategy, a Reporter Operon consisting of a promoter, RBS–reporter gene and terminator is inserted in several different phage genome locations and the optimal location is determined by the signal-to-noise ratio. (**B**) Strategy of Reporter Operon cloning leading to the isolation of 8M7 luminescent reporter phages. Different phage genome locations were selected for the insertion of the Reporter Operon by homologous-recombination-based cloning with phage targeting vectors (PTVs). Transformed KP2H7 bacteria carrying the PTVs are infected with 8M7 wildtype (WT) phages. Recombinant phages which carry the luminescent reporter operon emit a detectable signal upon phage interaction with the target bacterium.

For the Mcoc phage, the cloning strategy was to insert the Reporter Brick, comprised of *nluc* under the control of a strong ribosome binding site (RBS), directly downstream of the stop codon of the major capsid protein open reading frame (ORF). To promote successful homologous recombination in approximately 1% of phages, the phage targeting vector (PTV) was designed with roughly 500 bp of flanking sequences both 5’ (in the upstream homologous region (UHR)) and 3’ (in the downstream homologous region (DHR)) to the phage sequences of the insertion point (Figure 2A) [20,28,29,30,31]. The PTV was transformed into wildtype (WT) KP2H7 strain and KP2H7 bearing PTV plasmids were infected with wildtype phages, generating a mixture of recombinant and wildtype phage genomes. The mixture of recombinant and wildtype phages was enriched on WT KP2H7 again to ensure a detectable NanoLuc signal and determine the signal-to-noise ratio (SNR). This procedure yielded an SNR of >10 and the engineered phage was isolated by further enrichment steps, leading to progressive increases of the ratio of engineered phage to wildtype phage until the isolation of a single luminescent phage was possible [20].

The above method did not yield the desired result of an SNR > 10 with phage 8M7. Therefore, an alternative cloning strategy was employed. In this strategy, a strong promoter of the major capsid protein ORF was added upstream to the Reporter Brick unit followed by the major capsid protein ORF terminator. We called this unit Reporter Operon (Figure 2A). Because the Reporter Operon is an independent unit, the best genomic cloning location was determined by inserting this operon into five different genomic locations (Figure 2B). At each location a single ORF was used, inserting the Reporter Operon directly downstream of the stop codon while taking into account operon polarity [32]. The chosen regions encode the pore-forming tail tip protein, homing endonuclease, DNA polymerase, aerobic ribonucleoside diphosphate reductase large subunit, and a hypothetical protein. Upon comparison of the signal obtained at each of the five genomic locations, it was found that insertion of the Reporter Operon downstream of the homing endonuclease resulted in the highest SNR value of 290. This phage lysate was selected for further isolation of the engineered luminescent phage by an iterative process of enrichment until the luminescent phage was isolated (Figure 2B) [20].

### 2.2. Activity of Reporter Phages in Liquid Culture

**Figure 3:**
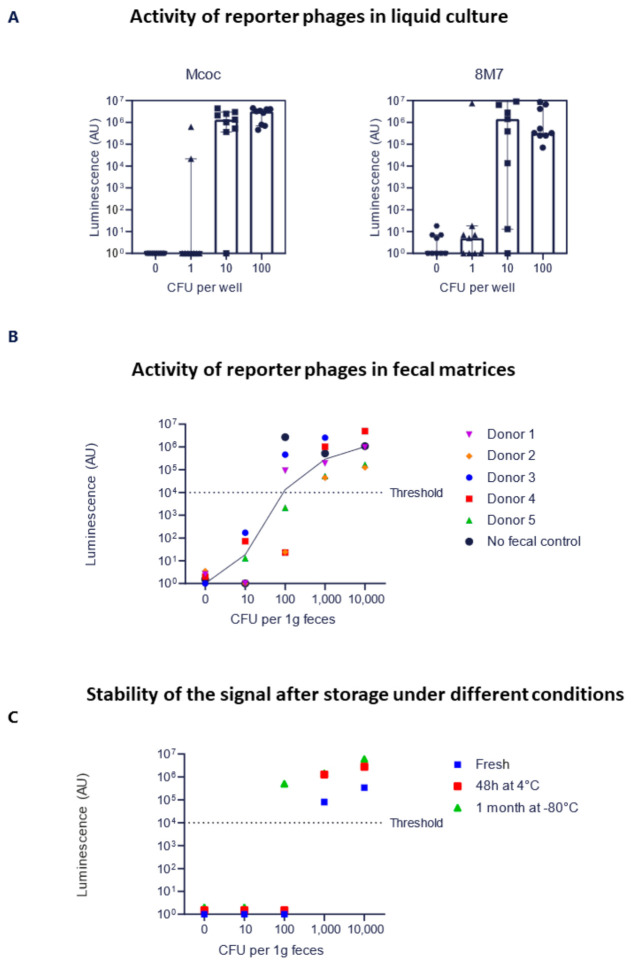
Limit of detection (LOD) assays. (**A**) Determination of the LOD of KP2H7 in liquid culture. Mcoc or 8M7 engineered luminescent phages were introduced into wells with 0–100 bacteria/well. Both phages successfully produced a high luminescence signal with 10 bacteria/well. Error bars represent standard error of the mean (S.E.M.) of nine independent experiments. (**B**) Luminescence detection in fecal samples spiked with KP2H7 bacteria. Fecal samples free of endogenous KP2H7 were spiked with known amounts of KP2H7 and infected with luminescent engineered Mcoc phage, followed by the detection of luminescence intensity. An amount of 100 bacteria in 100 mg feces was sufficient to produce a positive luminescence signal after phage infection. The trendline indicates the median results for each sample set. (**C**) Luminescence detection in fecal samples spiked with KP2H7 and stored under different conditions. Fecal samples that were spiked with defined amounts of KP2H7 bacteria were either stored at 4 °C for 48 h, −80 °C for one month, or examined when fresh. Mcoc engineered luminescent phages were then introduced to the samples and the luminescence signal was measured. Under all different storage conditions, 100 bacteria per 100 mg of feces was sufficient to produce a positive signal.

To demonstrate the sensitivity of the engineered Mcoc and 8M7 phages to their bacterial host strain, luminescence was tested in liquid bacterial cultures of KP2H7. Liquid cultures were inoculated with different numbers of bacterial cells per well (1, 10, and 100) in 200 µL BHIS and immediately infected with 10^3^ PFU of the luminescent phage 8M7 or Mcoc, followed by incubation for 3 h at 37 °C. After the incubation period, the luminescence signal was measured. The results demonstrated that as few as 10 initial bacterial cells was sufficient to achieve a median luminescence signal of 10^6^ arbitrary units (AU; Figure 3A). Since the cutoff for a positive signal was >10^4^ AU, this suggests a high level of sensitivity by both engineered reporter phages.

### 2.3. Activity of Reporter Phages in Fecal Matrices

Following the achievement of high sensitivity (>10^4^ AU) in liquid media measurements, the minimal number of KP2H7 cells that could be identified in fecal samples was determined.

Five fecal samples of 100 mg/mL BHIS were spiked with different numbers of target bacteria cells (1, 10, 100, and 1000). To overcome fecal inhibition of phage infection, a 1:10 dilution of the spiked fecal samples in BHIS was necessary. The results showed that a positive luminescence signal of >10^4^ AU was attained from 100 bacteria in 100 mg of fecal material homogenized in 1 mL of BHIS, which indicates a sensitivity of ≥1000 bacteria per 1 g of fecal matter (Figure 3B). This can be translated into a sensitivity above 1 part per million when taking into consideration the presence of another 10^11^ bacteria in 1 g of feces [33].

### 2.4. Stability of the Signal in Spiked Fecal Samples Stored under Different Conditions

To estimate the potential stability of fecal samples under different storage conditions, samples were spiked with a known number of bacteria and either tested immediately or following storage at 4 °C for 48 h or one month at −80 °C. We found that samples containing more than 1000 bacteria/g feces displayed a stable luminescence signal of >10^4^ AU across all conditions (Figure 3C), similar to the results found upon immediate testing (data not shown).

In conclusion, these results demonstrate that engineered luminescent phages are able to detect their target bacteria in fecal samples with high sensitivity. Furthermore, the ability to store the fecal samples while maintaining bacterial load integrity enables parallel detection across multiple samples that were acquired at varying times, thus offering a promising tool to advance the application of phage therapy in a precise manner.

## 3. Discussion

Despite being crucial for effective diagnosis and disease management by phage therapy, the rapid detection and reliable identification of phage-sensitive bacterial pathogens remain challenges for the clinical laboratory. Rapid, potentially on-site, bacterial diagnostic testing using luminescent engineered phages is a culture- and isolation-free approach to detect the presence of phage-sensitive bacterial colonization in clinical samples, including feces. The rapid evaluation of target bacteria susceptibility could guide physicians in decision-making and provide a realistic path for precision treatment that will ensure a high level of efficacy with phage therapy and overcome one of the greatest challenges in treating microbiome-related diseases.

Luminescent phages have previously been engineered to detect bacterial cells ranging from *Escherichia coli* to *Yersinia pestis* present in different matrices (Table 1). The reporter phage assays use different quantification methods to measure system sensitivity, so comparisons between the different approaches are not straightforward. Zhang et al. reported the insertion of NanoLuc into the lysogenic bacteriophage ɸV10 for the detection of *E. coli* O157:H7 [34]. Hinkley et al. described the development of an assay to detect *E. coli* using engineered T7 bacteriophages with NanoLuc [35]. Both research groups focused on the detection of contaminants in food and drinking water matrices. Studies that have been described in the published literature using different luminescence systems to examine different matrices, including clinical samples such as blood and serum, are listed in Table 1.

The present study describes the cloning of the luciferase gene *nluc* into the genomes of two phages, Mcoc and 8M7, comprising part of a therapeutic cocktail for the treatment of IBD caused by a pro-inflammatory *KP* bacterial strain, KP2H7. The resulting reporter phages are intended to detect target bacteria sensitive to infection by at least one of these phages to determine patient eligibility for treatment. In this work, two different cloning strategies employing different reporter gene structures and genome insertion sites were required to successfully develop the two reporter phages.

While initial studies (unpublished data) demonstrated very high sensitivity by both reporter phages to detect target bacteria in culture media, the current studies were focused on examining the activity of the assay in a relevant clinical matrix. The relevant clinical sample for detecting the presence of the pro-inflammatory KP2H7 in the gut of IBD patients is fecal matter. As fecal samples are not constant in composition and do not constitute a defined matrix due to the variation in their physiochemical and bacterial composition, studies using this matrix were carried out with five different fecal samples known to be free of endogenous KP2H7 [41]. The reporter phages were able to detect spiked KP2H7 in all five fecal samples with very high specificity, as they did not produce a signal in unspiked samples. These results confirm the potential of using similarly engineered reporter phages to sensitively detect the presence of target bacteria directly in fecal samples and are clinically relevant when interrogating bacteria in the gut.

In order to understand the need for immediate performance of the assay on fresh fecal samples, we investigated the effects of different fecal storage conditions on the KP2H7 signal obtained with the reporter phage. An amount of 1000 bacteria/1 g feces was shown to result in a detectable luminescence signal under all storage conditions and durations tested. This number is 100-fold less than the average bacterial load of KP2H7 observed in IBD patients who were shown to be colonized by this bacteria using a proprietary double qPCR method (not shown), thus making this assay clinically relevant. The ability to store fecal samples without major losses in the reporter-phage-based detection of bacterial pathogens is advantageous, as it enables sample transfer and collection to allow simultaneous high-throughput sample processing.

These combined results demonstrate the potential of luminescent reporter phages and the need for flexibility in carrying out the engineering of different phages to achieve the greatest sensitivity. The reported findings expand the possibilities for the use of luminescent reporter phages in phage-susceptibility diagnostics by providing additional engineering methods for deriving sensitive reporter phages. They also demonstrate the feasibility of directly testing fecal samples, provided that the fecal matter is diluted. One limitation of this study is that the number of different fecal samples that were tested was relatively small. Larger sample size could better determine the reliability of the described method in multiple fecal matrices. Another constraint is the requirement in the current methodology of two incubation periods. Future optimization of the assay should enable faster turnaround time, which will further enhance the promise of this approach to provide rapid on-site or point-of-care co-diagnostics as an integral method to guide the growing field of chronic disease treatment, particularly of diseases associated with gut microbiome imbalances, using phage therapy in a precision medicine approach.

## 4. Materials and Methods 

### 4.1. Stool and Sewage Samples

Stool samples were collected at the Tel Aviv Sourasky Medical Center under approval of the Institutional Review Board dated September 2019 (IRB approval number 0367-15-TLV) and iSpecimen, Inc. (Lexington, MA, USA). 

Reporter phages Mcoc and 8M7 were isolated in-house from sewage samples. Phages sequences can be found in the Supplementary Material Section, Mcoc sequence File S1, 8M7 sequences File S2.

### 4.2. Klebsiella pneumonia Bacterial Strain

*KP* strain KP2H7 was kindly provided by Dr. Kenya Honda (Department of Microbiology and Immunology, Keio University School of Medicine, Tokyo, Japan) [4].

### 4.3. Polymerase Chain Reaction (PCR)

Primers were synthesized by Sigma-Aldrich (Rehovot, Israel), Primers list used in this study can be found in the Supplementary Materials Section- Supplementary File S3. The NanoLuc gene fragment was synthesized de novo by Integrated DNA Technologies, Inc. (Belgium). PCR was performed using PrimeSTAR Max DNA Polymerase (Takara, Japan). Cloning procedures were designed using Clone Manager 9 Professional Edition (Scientific & Educational Software, USA). pCR™-Blunt plasmid (ThermoFisher Scientific, USA) was used as the template for PCR reactions. Gibson Assembly^®^ Master Mix (NEB, USA) was used for the DNA assembly reaction. The resulting recombinant NanoLuc phage targeting vectors were purified from *E. coli* DH5α cultures using the Presto™ Mini Plasmid Kit (PDH300) (Geneaid Biotech Ltd., New Taipei City, Taiwan) and transformed into KP2H7 by electroporation [42].

### 4.4. Recombinant Phage Mixture Generation

KP2H7 bacteria bearing the phage targeting vector (PTV) were grown to mid-log phase in lysogeny broth (LB) with 50 µg/mL kanamycin (Sigma-Aldrich, Rehovot, Israel) until an optical density (OD) of 0.2 was achieved. At OD ~0.2, recombinant KP2H7 were infected with wildtype phages at a multiplicity of infection (MOI_input_) of 0.01 and incubated for 16 h at 37 °C to allow for phage amplification [43]. The lysate was then centrifuged at 9000× *g* and syringe filtered (0.22 µm; Merck, Germany). This resulted in a mix of wildtype and recombinant phages that were used in further enrichment steps.

### 4.5. Recombinant Phage Isolation

KP2H7 bacteria were grown to mid-log phase (OD ~ 0.2) and infected with the mixture of recombinant and wildtype phages (first enrichment). Phages were enriched until a pure recombinant phage culture was obtained following the protocol of Brownell et al. [20]. Luminescence was measured as previously described [20]. The ratio of engineered phages expressing the bioluminescent reporter gene *nluc* to wildtype phages was determined using the Nano-Glo^®^ assay (Promega, WI, USA) and SPARK^®^ Multimode Microplate Reader (Tecan, Switzerland) according to the manufacturer’s instructions. For clone sequence confirmation we used the outsourcing services of Hy Laboratories Ltd. (Israel). The Illumina MiSeq technology (paired-end, 250 base pair runs, 50×) and the Illumina Nextera protocol were applied [44]. Phage genome sequences were assembled de novo using plasmid SPAdes [45]. Enrichment cycles were carried out until the ratio between wildtype phage and engineered phage was about 10:1 [20]. From these enriched populations, a single luminescent plaque was picked and propagated to form the luminescent bacteriophage working stock.

### 4.6. Signal-To-Noise Ratio (SNR) Determination

To calculate the signal-to-noise ratio, we compared the luciferase signal between the lysate of the recombinant phage mixture and the phages that are obtained after the first enrichment. Despite the lack of bacterial promoter, the recombinant phage mixture had a luminescent signal due to the background expression of NanoLuc from the PTV plasmid. Therefore, this luminescent signal was defined as the baseline, or “noise”, and any luminescent signal that was at least 10 times higher was indicative of the presence of engineered luminescent phages with sufficient NanoLuc expression. 

### 4.7. Bacterial Preparation for Fecal Spiking

The starter culture was prepared by inoculating 2 to 3 cell colonies of KP2H7 into 4 mL Gibco™ Bacto™ Brain Heart Infusion Supplement (BHIS) (Life Technologies Cooperation, MI, USA) and incubating aerobically overnight (O/N) in a shaking incubator at 37 °C. The bacterial starter cultures were diluted 10^−1^ to 10^−9^ in filtered BHIS in preparation for spiking into fecal samples. Colony forming units (CFU) were measured to determine bacterial number in each dilution and the values obtained were rounded up to the next round number value, thus sometimes underestimating actual sensitivity. 

### 4.8. Phage Sensitivity Assay in Liquid

Bacteria were prepared as described above. Dilutions of 10^−4^, 10^−5^, 10^−6^, 10^−7^, and 10^−8^ were prepared. An amount of 200 µL of each dilution was transferred into 9 wells of a 96-well plate. Mcoc or 8M7 reporter phages at a titer of 10^3^ PFU in phage buffer (10 mM Tris, pH 7.5, 10 mM MgCl_2_, 68 mM NaCl, 1 mL CaCl_2_) were added to the bacteria. To promote a better infection rate, divalent ions MnCl_2_, CaCl_2_, and MnCl_2_ were added to a final concentration of 1 mM each. Plates were incubated for 3 h in a shaking incubator at 37 °C. After the incubation, the samples were centrifuged at 2200× *g* for 20 min at 4 °C; 100 µL from each sample was transferred and mixed with 100 µL of Nano-Glo^®^ assay mix. The luminescence signal was detected using SPARK^®^ Multimode Microplate Reader. Values of the signal for each sample can be found in Supplementary File S4.

### 4.9. Sample Processing and Phage Sensitivity Assay in Fecal Matrices

Fecal samples from five different healthy donors were shown by specific qPCR not to contain endogenous KP2H7 sequences (not shown) and then spiked with known numbers of the target bacterial strain and infected with Mcoc reporter phages to examine their ability to detect KP2H7. All samples were run in biological and technical triplicates. Five aliquots were prepared per sample, each containing 100 mg of feces. Each aliquot was resuspended in 1 mL of filtered BHIS and added into 1 mL BHIS in a homogenization tube (total of 2 mL per sample). Each aliquot was spiked with a known amount of KP2H7 bacterial cells ranging from 1 to 1000 cells. To promote a better infection rate, divalent ions were added as above. All dilutions were homogenized using gentle MACS™ Octo Dissociator (Miltenyi Biotec, Germany) to obtain a homogeneous distribution of spiked bacteria. Following homogenization, each tube was further diluted 1:10 with BHIS to reduce fecal inhibition of phage infection. Bacterial samples were incubated under aerobic conditions in a shaking incubator at 37 °C for 5–7 h. Mcoc reporter phages at a titer of 10^4^ PFU were added to the sample in phage buffer and incubated O/N in a shaking incubator at 37 °C. Samples were centrifuged at 2200× *g* for 20 min at 4 °C. Samples of 100 µL each were mixed with 100 µL of Nano-Glo^®^ assay mix. The luminescence signal was detected using a SPARK^®^ Multimode Microplate Reader. Values of the signal for each sample can be found in Supplementary file S4.

### 4.10. Determination of Arbitrary Unit Cutoff for Positive Signal 

To determine the cut-off value for a positive signal, a training set consisting of 108 positive (spiked) and 108 known negative samples was generated. From luminescent measurements carried out on this dataset, two ranges of values were obtained, one for the negative samples and one for the positive samples. These ranges were observed to overlap. A value within this overlap region was selected such that it gave the smallest number of false positives and false negatives. A threshold of 10,000 arbitrary units was obtained, in which the false negative ratio was 0.204 and the false positive ratio was 0.111.

## Figures and Tables

**Table 1:** Reporter phages described in the literature compared with the here-described phages.

Publication	Target Bacteria	Matrix	Reporter Phage and Detection Method	Sensitivity	Stability of Signal
Present	*Klebsiella pneumonia* strain (KP2H7)	Feces (requires dilution of 1:10 to overcome presence of inhibitors) which allows diagnostics of gut microbiome in chronic diseases	Mcoc and 8M7 NanoLuc	100 cells per 100 mg feces	Possibility of both on-site POC diagnostics and diagnostics by a central laboratory. Useful diagnostic assay with the potential for field application
Zhang et al. [34]	*E. coli* O157:H7	Food matrix incl. ground beef	ɸV10 NanoLuc	Detection of a very low quantity of contaminating *E. coli* O157:H7 (5–6 cells) in 7–9 h	N/A
Hinkley et al. [35]	*E. coli* ECOR13	Drinking water	T7 NanoLuc	Identification of less than 20 colony forming units (CFU) *E. coli* in 100 mL drinking water within 5 h (0.2CFU/mL)	N/A
Gupta et al. [36]	*Brucella abortus; Brucella melitensis*	Clinical samples—aborted cattle fetus stomach contents	Brucella phage Luciferase	Average increase of luminescence was 10.03 fold	Useful diagnostic assay with the potential for field application
Schofield et al. [37]	*Bacillus anthracis*	Blood samples	Wβ luxAB	10^5^ CFU/mL	N/A
Schofield et al. [38]	*Yersenia pestis*	Rapid diagnostic detection of cultivated *Y. pestis* isolates or infected clinical serum specimens	φA1122 luxAB	10^3^ CFU/mL within 60min	
Willford et al. [39]	Shiga toxin producing *E. coli*	Food; drinking water	Phazyme Enzyme-labeled phage	10^5^–10^6^ CFU/mL in pure culture	In a simple and rapid manner, with minimal need for instrumentation to interpret the test result
Franche et al. [40]	Enterobacteriaceae	Water	HK620; HK97; GFP	10^4^ bacteria/mL in 1.5 h	Neither concentration nor enrichment step required

## Data Availability

Data supporting reported results can be found at the Supplementary Materials Section.

## Supplementary Materials

The following are available online at https://www.mdpi.com/article/10.3390/ph14040347/s1, File S1. Mcoc phage sequence, File S2. 8M7 phage sequence, File S3 Primers list used in this study, File S4. luminescence data.

## References

[1] Uemura N., Okamoto S., Yamamoto S., Matasumura N., Yamaguchi S., Yamakido M., Taniyama K., Sasaki N., Schlemper R.J. (2001). *Helicobacter pylori* infection and the development of gastric cancer. N. Engl. J. Med..

[2] Kostic A.D., Chun E., Robertson L., Glickman J.N., Gallini C.A., Michaud M., Clancy T.E., Chung D.C., Lochhead P., Hold G.L. (2013). *Fusobacterium nucleatum* potentiates intestinal tumorigenesis and modulates the tumor immune microenvironment. Cell Host Microbe.

[3] Paczosa M.K., Mecsas J. (2016). *Klebsiella pneumoniae*: Going on the offense with a strong defense. Microbiol. Mol. Biol. Rev..

[4] Atarashi K., Suda W., Luo C., Kawaguchi T., Motoo I., Narushima S., Kiguchi Y., Yasuma K., Watanabe E., Tanoue T. (2017). Ectopic colonization of oral bacteria in the intestine drives TH1 cell induction and inflammation. Science.

[5] Francino M.P. (2016). Antibiotics and the human gut microbiome: Dysbioses and accumulation of resistances. Front. Microbiol..

[6] Jernberg C., Löfmark S., Edlund C., Jansson J.K. (2010). Long-term impacts of antibiotic exposure on the human intestinal microbiota. Microbiology.

[7] Combarros-Fuertes P., Fresno J.M., Estevinho M.M., Sousa-Pimenta M., Tornadijo M.E., Estevinho L.M. (2020). Honey: Another alternative in the fight against antibiotic-resistant bacteria?. Antibiotics.

[8] Rossiter S.E., Fletcher M.H., Wuest W.M. (2017). Natural products as platforms to overcome antibiotic resistance. Chem. Rev..

[9] Morehead M.S., Scarbrough C. (2018). Emergence of global antibiotic resistance. Prim. Care Clin. Off. Pract..

[10] Ndagi U., Falaki A.A., Abdullahi M., Lawal M.M., Soliman M.E. (2020). Antibiotic resistance: Bioinformatics-based understanding as a functional strategy for drug design. RSC Adv..

[11] Payne R.J., Phil D., Jansen V.A. (2000). Phage therapy: The peculiar kinetics of self-replicating pharmaceuticals. Clin. Pharmacol. Ther..

[12] Nobrega F.L., Vlot M., de Jonge P.A., Dreesens L.L., Beaumont H.J.E., Lavigne R., Dutilh B.E., Brouns S.J.J. (2018). Targeting mechanisms of tailed bacteriophages. Nat. Rev. Microbiol..

[13] Ács N., Gambino M., Brøndsted L. (2020). Bacteriophage enumeration and detection methods. Front. Microbiol..

[14] Kropinski A.M., Mazzocco A., Waddell T.E., Lingohr E., Johnson R.P., Clokie M.R.J., Kropinski A.M. (2009). Enumeration of bacteriophages by double agar overlay plaque assay. Bacteriophages: Methods and Protocols, Volume 1: Isolation, Characterization, and Interactions.

[15] Henry M., Biswas B., Vincent L., Mokashi V., Schuch R., Bishop-Lilly K.A., Sozhamannan S. (2012). Development of a high throughput assay for indirectly measuring phage growth using the OmniLogTM system. Bacteriophage.

[16] Lammens E.-M., Nikel P.I., Lavigne R. (2020). Exploring the synthetic biology potential of bacteriophages for engineering non-model bacteria. Nat. Commun..

[17] Lemire S., Yehl K.M., Lu T.K. (2018). Phage-based applications in synthetic biology. Annu. Rev. Virol..

[18] Loessner M.J., Rudolf M., Scherer S. (1997). Evaluation of luciferase reporter bacteriophage A511::LuxAB for detection of *Listeria monocytogenes* in contaminated foods. Appl. Environ. Microbiol..

[19] Loessner M.J., Rees C.E., Stewart G.S., Scherer S. (1996). Construction of luciferase reporter bacteriophage A511::LuxAB for rapid and sensitive detection of viable *Listeria* cells. Appl. Environ. Microbiol..

[20] Brownell D., King J., Caliando B., Sycheva L., Koeris M., Clokie M.R.J., Kropinski A., Lavigne R. (2019). Engineering bacteriophage-based biosensors. Bacteriophages.

[21] Meile S., Sarbach A., Du J., Schuppler M., Saez C., Loessner M.J., Kilcher S. (2020). Engineered reporter phages for rapid bioluminescence-based detection and differentiation of viable *Listeria* cells. Appl. Environ. Microbiol..

[22] Shin J., Jardine P., Noireaux V. (2012). Genome replication, synthesis, and assembly of the bacteriophage T7 in a single cell-free reaction. ACS Synth. Biol..

[23] Ando H., Lemire S., Pires D.P., Lu T.K. (2015). Engineering modular viral scaffolds for targeted bacterial population editing. Cell Syst..

[24] Kilcher S., Studer P., Muessner C., Klumpp J., Loessner M.J. (2018). Cross-genus rebooting of custom-made, synthetic bacteriophage genomes in L-form bacteria. Proc. Natl. Acad. Sci. USA.

[25] Davis J.J., Wattam A.R., Aziz R.K., Brettin T., Butler R., Butler R.M., Chlenski P., Conrad N., Dickerman A., Dietrich E.M. (2020). The PATRIC Bioinformatics Resource Center: Expanding data and analysis capabilities. Nucleic Acids Res..

[26] Brettin T., Davis J.J., Disz T., Edwards R.A., Gerdes S., Olsen G.J., Olson R., Overbeek R., Parrello B., Pusch G.D. (2015). RASTtk: A Modular and extensible implementation of the RAST algorithm for building custom annotation pipelines and annotating batches of genomes. Sci. Rep..

[27] Bankevich A., Nurk S., Antipov D., Gurevich A.A., Dvorkin M., Kulikov A.S., Lesin V.M., Nikolenko S.I., Pham S., Prjibelski A.D. (2012). SPAdes: A new genome assembly algorithm and its applications to single-cell sequencing. J. Comput. Biol..

[28] Tao P., Wu X., Tang W.-C., Zhu J., Rao V. (2017). Engineering of bacteriophage T4 genome using CRISPR-Cas9. ACS Synth. Biol..

[29] Hall M.P., Unch J., Binkowski B.F., Valley M.P., Butler B.L., Wood M.G., Otto P., Zimmerman K., Vidugiris G., Machleidt T. (2012). Engineered luciferase reporter from a deep sea shrimp utilizing a novel imidazopyrazinone substrate. ACS Chem. Biol..

[30] Drulis-Kawa Z., Mackiewicz P., Kęsik-Szeloch A., Maciaszczyk-Dziubinska E., Weber-Dąbrowska B., Dorotkiewicz-Jach A., Augustyniak D., Majkowska-Skrobek G., Bocer T., Empel J. (2011). Isolation and characterisation of KP34—A novel ΦKMV-like bacteriophage for *Klebsiella pneumoniae*. Appl. Microbiol. Biotechnol..

[31] Pouillot F., Blois H., Iris F. (2010). Genetically engineered virulent phage banks in the detection and control of emergent pathogenic bacteria. Biosecur. Bioterror..

[32] Levin-Karp A., Barenholz U., Bareia T., Dayagi M., Zelcbuch L., Antonovsky N., Noor E., Milo R. (2013). Quantifying translational coupling in *E. coli* synthetic operons using RBS modulation and fluorescent reporters. ACS Synth. Biol..

[33] Sender R., Fuchs S., Milo R. (2016). Revised estimates for the number of human and bacteria cells in the body. PLoS Biol..

[34] Zhang D., Coronel-Aguilera C.P., Romero P.L., Perry L., Minocha U., Rosenfield C., Gehring A.G., Paoli G.C., Bhunia A.K., Applegate B. (2016). The use of a novel NanoLuc-based reporter phage for the detection of *Escherichia coli* O157:H7. Sci. Rep..

[35] Hinkley T.C., Garing S., Jain P., Williford J., Le Ny A.-L.M., Nichols K.P., Peters J.E., Talbert J.N., Nugen S.R. (2020). A Syringe-based biosensor to rapidly detect low levels of *Escherichia coli* (ECOR13) in drinking water using engineered bacteriophages. Sensors.

[36] Gupta V., Saxena H.M. (2017). A new bacteriophage based luminescence assay for diagnosis of brucellosis. Indian J. Exp. Biol..

[37] Schofield D.A., Sharp N.J., Vandamm J., Molineux I.J., Spreng K.A., Rajanna C., Westwater C., Stewart G.C. (2013). *Bacillus anthracis* diagnostic detection and rapid antibiotic susceptibility determination using ‘bioluminescent’ reporter phage. J. Microbiol. Methods.

[38] Schofield D.A., Molineux I.J., Westwater C. (2009). Diagnostic bioluminescent phage for detection of yersinia pestis. J. Clin. Microbiol..

[39] Willford J.D., Bisha B., Bolenbaugh K.E., Goodridge L.D. (2011). Luminescence based enzyme-labeled phage (phazyme) assays for rapid detection of shiga toxin producing *Escherichia coli* serogroups. Bacteriophage.

[40] Franche N., Vinay M., Ansaldi M. (2017). Substrate-independent luminescent phage-based biosensor to specifically detect enteric bacteria such as *E. coli*. Environ. Sci. Pollut. Res..

[41] Rose C., Parker A., Jefferson B., Cartmell E. (2015). The characterization of feces and urine: A review of the literature to inform advanced treatment technology. Crit. Rev. Environ. Sci. Technol..

[42] Nováková J., Izsáková A., Grivalský T., Ottmann C., Farkašovský M. (2014). Improved method for high-efficiency electrotransformation of *Escherichia coli* with the large BAC plasmids. Folia Microbiol..

[43] Abedon S.T. (2016). Phage therapy dosing: The problem(s) with Multiplicity of Infection (MOI). Bacteriophage.

[44] Baym M., Kryazhimskiy S., Lieberman T.D., Chung H., Desai M.M., Kishony R. (2015). Inexpensive multiplexed library preparation for megabase-sized genomes. PLoS ONE.

[45] Antipov D., Hartwick N., Shen M., Raiko M., Lapidus A., Pevzner P.A. (2016). PlasmidSPAdes: Assembling plasmids from whole genome sequencing data. Bioinformatics.

